# Cannabidiol for the Treatment of Brain Disorders: Therapeutic Potential and Routes of Administration

**DOI:** 10.1007/s11095-023-03469-1

**Published:** 2023-01-12

**Authors:** Grace Tsz Yan Yau, Waiting Tai, Jonathon Carl Arnold, Hak-Kim Chan, Philip Chi Lip Kwok

**Affiliations:** 1 Advanced Drug Delivery Group, School of Pharmacy, Faculty of Medicine and Health, The University of Sydney, Sydney, NSW 2006 Australia; 2 Lambert Initiative for Cannabinoid Therapeutics, Brain and Mind Centre, The University of Sydney, Sydney, NSW 2050 Australia; 3 Discipline of Pharmacology, School of Pharmacy, Faculty of Medicine and Health, The University of Sydney, Sydney, NSW 2006 Australia

**Keywords:** brain disorders, cannabidiol, inhalation, pharmacokinetics, systemic delivery

## Abstract

The use of cannabidiol (CBD) for treating brain disorders has gained increasing interest. While the mechanism of action of CBD in these conditions is still under investigation, CBD has been shown to affect numerous different drug targets in the brain that are involved in brain disorders. Here we review the preclinical and clinical evidence on the potential therapeutic use of CBD in treating various brain disorders. Moreover, we also examine various drug delivery approaches that have been applied to CBD. Due to the slow absorption and low bioavailability with the current oral CBD therapy, more efficient routes of administration to bypass hepatic metabolism, particularly pulmonary delivery, should be considered. Comparison of pharmacokinetic studies of different delivery routes highlight the advantages of intranasal and inhalation drug delivery over other routes of administration (oral, injection, sublingual, buccal, and transdermal) for treating brain disorders. These two routes of delivery, being non-invasive and able to achieve fast absorption and increase bioavailability, are attracting increasing interest for CBD applications, with more research and development expected in the near future.

## Introduction

The medical use of cannabis (*Cannabis sativa*) can be traced to more than 4000 years ago in China [1]. Much recent research has been conducted to explore and expand its therapeutic application. The two major phytocannabinoids in cannabis are delta-9-tetrahydrocannabinol (THC) and cannabidiol (CBD). The therapeutic use of CBD is more attractive than THC due to its non-psychoactive properties [2]. This has led to an increasing interest in using CBD to treat brain disorders, such as epilepsy, pain, depression, anxiety, and psychosis [3–10]. This is reflected in the approval of Epidiolex® (a CBD oral solution) by the United States Food and Drug Administration in June 2018 for treating refractory epileptic seizures in patients with Dravet and Lennox-Gastaut syndromes, as well as in July 2020 for seizures associated with tuberous sclerosis complex [11].

Different administration routes of CBD have been explored to better understand its pharmacokinetics (PK) and to provide information on optimal route/s for the systemic delivery of CBD for treating brain disorders. These include intravenous, oral, sublingual, buccal, pulmonary, intranasal, transdermal routes, and more. The bioavailability of CBD depends on the route of administration [12]. CBD has low aqueous solubility (12.6 mg/L) and low bioavailability (4–20%) due to extensive first pass metabolism [13–15]. The poor oral bioavailability leads to low drug efficiency, hence a higher dose is required to reach the therapeutic concentration, which subsequently increases the risk of side effects and drug interactions [16]. Although oral administration route is most commonly used, a better alternative route that increases bioavailability is needed to deliver CBD systemically.

Understanding the PK properties of CBD is critical to its development and clinical use in the future. This review aims to present an overview of the therapeutic use of CBD for brain conditions and the PK profiles of various CBD delivery routes in animal and human studies, followed by discussing the most suitable routes of administration to optimize CBD delivery for brain disorders.

## Cannabidiol (CBD)

### Mechanism of Action

The mechanism of action of CBD in neurological and psychiatric conditions is still being investigated, however, several central nervous system (CNS) drug targets are implicated in its actions including the modulation of the brain natural cannabis system, the endocannabinoid system [17].

Two main cannabinoid (CB) receptors were identified in the early 1990s [17]. CB1 receptors are located in the CNS, spinal cord, and peripheral nervous system, but also in peripheral tissues, including the endocrine glands, heart, as well as the reproductive, urinary, and gastrointestinal tracts [17]. CB2 receptors are found primarily in the immune system, such as the leucocytes, spleen, and tonsils [17]. The ligands of CB1 and CB2 receptors can be endogenous (e.g. anandamide and 2-arachidonoylglycerol) or exogenous, such as phytocannabinoids (e.g. THC and CBD) [18]. CBD has a low affinity to CB receptors and does not activate CB1 and CB2 receptors, leading to the lack of psychotropic activity [19, 20]. However, despite the low affinity, CBD is a negative allosteric modulator of CB receptors [21–24] and antagonizes CB1/CB2 agonists [25]. CBD may also act as an indirect cannabinoid receptor agonist – CBD has been shown to increase tissue concentrations of anandamide via inhibition of its degradative enzyme fatty acid amide hydrolase or by inhibiting transport mediated by fatty acid binding proteins [26, 27]. Indeed, human clinical studies have shown CBD increases plasma concentrations of anandamide [28].

CBD also acts on various non-endocannabinoid receptors (Table I), including but not limited to G protein-coupled receptors (GPR3, GPR6, GPR12, GPR55) [22, 29–32], transient receptor potential channels (TRPM8, TRPA1, TRPV1, TRPV2) [27, 33–36], serotonin receptors [37, 38], mu- and delta-opioid receptors [39, 40], peroxisome proliferator-activated receptor gamma [41–43], and glycine receptors [44, 45]. CBD was shown to act as a positive allosteric modulator of the inhibitory GABA_A_ receptor and that its effects on potentiating this channel were complementary to the effects of the benzodiazepine and anticonvulsant clobazam [46, 47]. Interaction with multiple receptors leads to future discovery and potential therapeutic use of CBD in different CNS conditions [39, 48] (Table I).

**Table I:** Major Receptor Targets of CBD and their Potential Indications in Brain Disorders

Receptors	K_i_ (µM)	EC_50_/ IC_50_ (µM)	CBD activity	Potential indications [39, 48]	References
CB1	2.01	> 30	Negative allosteric modulator	Pain, cardiovascular diseases	[21–23, 40]
CB2	ND	> 30	Negative allosteric modulator	Pain	[22, 24]
GPR3	ND	1	Inverse agonist	Neuropathic pain, neurodegenerative diseases	[30]
GPR6	ND	0.1	Inverse agonist	Neuropathic pain, neurodegenerative diseases	[30]
GPR12	ND	10	Inverse agonist	Neuropathic pain, neurodegenerative diseases	[29, 30]
GPR55	ND	0.445	Antagonist	Epilepsy, neurodegenerative diseases	[22, 31, 32]
TRPM8	ND	0.06 – 0.14	Antagonist	Pain	[27, 33]
TRPA1	ND	0.096 – 0.11	Agonist	Pain, inflammation	[27, 33]
TRPV1	ND	1 – 3.5	Agonist	Epilepsy, pain, inflammation, neurodegenerative diseases, psychosis	[27, 34]
TRPV2	ND	1.25 – 31.7	Agonist	Epilepsy, pain, inflammation	[27, 35, 36]
5-HT_1A_	16	ND	Agonist	Epilepsy, movement disorders, depression, anxiety, psychosis	[37, 38]
μ-opioid	31.6	10	Positive allosteric modulator	Neurodegenerative diseases, addiction	[40]
δ-opioid	18.4	10.7	Positive allosteric modulator	Neurodegenerative diseases, addiction	[40]
PPAR-γ	ND	20.1	Agonist	Neurodegenerative diseases, inflammation	[42, 43]
α1-glycine	ND	12.3	Positive allosteric modulator	Neurodegenerative diseases, inflammation	[44]
		132.4	Agonist		
α1β-glycine	ND	18.1	Positive allosteric modulator	Neurodegenerative diseases, inflammation	[44]
		144.3	Agonist		
α3-glycine	ND	3	Agonist	Neuropathic pain, inflammation	[45]
GABA_A_	ND	0.9 – 23.1	Positive allosteric modulator	Epilepsy, anxiety, insomnia	[47]
FAAH	ND	15.2 – 27.5	Inhibitor	Epilepsy, psychosis, insomnia	[27, 34]

Abbreviations: 5-HT_1A_, Serotonin receptor 1A; CB, Cannabinoid receptor; FAAH, Fatty acid amide hydrolase; GABA_A_, γ-aminobutyric acid type A receptor; GPR, G protein-coupled receptor; ND, not determined; PPAR-γ, Peroxisome proliferator-activated receptor gamma; TRPA1, TRP channel of ankyrin type 1; TRPM8, TRP channel of melastatin type-8; TRPV1, TRP channel of vanilloid type-1; TRPV2, TRP channel of vanilloid type-2

### Therapeutic Effect of CBD on Brain Disorders

As CBD can interact with many targets in the CNS, animal and human studies were conducted to evaluate the therapeutic potentials of CBD in different conditions [49–51] (Table II). Its antiepileptic [3, 52], analgesic [53, 54], neuroprotective [55–58], antidepression [59, 60], anxiolytic [60, 61], antipsychotic [28, 62], and sedative effects [63, 64] were studied and might be useful in treating brain disorders.

**Table II:** Therapy Studies Evaluating Clinical Response of CBD in Animals and Patients with Different Medical Conditions

References	Study Design	Subjects	Treatment	Outcomes
*Epilepsy*				
Devinsky *et al*., 2017 [3]	Randomized, double-blind, placebo-controlled study	Children and young adults with Dravet syndrome and drug-resistant seizures (n = 120)	20 mg/kg CBD (p.o.) or placebo daily for 14 weeks, in addition to standard antiepileptic treatment	Reduction in frequency of convulsive seizures by 38.9% per month in CBD group, as compared to 13.3% in placebo group; Overall patients’ condition, assessed with CGIC, improved in 62% of CBD group, as compared to 34% of placebo group
Scheffer *et al*., 2021 [68]	Open-label extension study	Subjects with Dravet syndrome who completed in previous studies (n = 315)	20–30 mg/kg CBD (p.o.) daily based on response and tolerability, in addition to standard antiseizure medications	Long term use of CBD reduced monthly convulsive (45–74%) and total seizures (49–84%) frequency for up to 156 weeks; Improvement in overall condition in more than 83% of patients, assessed with S/CGIC
Devinsky *et al*., 2018 [52]	Phase 3, randomized, double-blind, placebo-controlled study	Subjects with Lennox-Gastaut syndrome (n = 225)	10, 20 mg/kg CBD (p.o.) or placebo in two equally divided doses daily for 14 weeks, in addition to standard antiepileptic treatment	Reduction in drop-seizure frequency by 41.9% and 37.2% in 20 and 10 mg CBD group respectively, as compared to 17.2% in placebo group; Overall patients’ condition, assessed with S/CGIC, improved in 57% (20 mg) and 66% (10 mg) of CBD group, as compared to 44% of placebo group
Patel *et al*., 2021 [69]	Open-label extension study	Subjects with Lennox-Gastaut syndrome who completed in previous studies (n = 366)	20–30 mg/kg CBD (p.o.) daily based on response and tolerability, in addition to standard antiseizure medications	Long term use of CBD resulted in sustained reduction in drop (48–71%) and total seizures (48–68%) frequency for up to 156 weeks; Improvement in overall condition in more than 87% of patients, assessed with S/CGIC
Hess *et al*., 2016 [4]	Expanded-access study	Subjects with treatment-resistant epilepsy associated with tuberous sclerosis complex (n = 18)	Maximum of 50 mg/kg CBD (p.o.) or placebo daily for 12 months	Reduction in weekly seizure frequency by 48.8% after 3 months of CBD treatment; Improvements in cognitive and behavioral function in 85.7% and 66.7% of patients respectively
Thiele *et al*., 2021 [67]	Phase 3, randomized, double-blind, placebo-controlled parallel group study	Subjects with seizures associated with tuberous sclerosis complex (n = 224)	25 or 50 mg/kg CBD (p.o.) or placebo daily for 16 weeks	Reduction in seizures associated with tuberous sclerosis complex by 48.6% and 47.5% in 25 and 50 mg CBD group respectively, as compared to 26.5% in placebo group
Thiele *et al*., 2022 [70]	Open-label extension study	Subjects with seizures associated with tuberous sclerosis complex (n = 199)	25–50 mg/kg CBD (p.o.) daily based on response and tolerability	Long term use of CBD reduced seizures associated with tuberous sclerosis complex by 54–68% and sustained through 48 weeks of treatment; Improvement in overall condition in 87% of patients at 26 weeks, assessed with S/CGIC
*Pain*				
Brioschi *et al*., 2020 [54]	Animal study	Client-owned dogs with osteoarthritis and associated signs of joint dysfunction (n = 21)	2 mg/kg of oral transmucosal CBD oil every 12 h or only controlled drugs (firocoxib or prednisone) for 12 weeks	A decrease in Pain Severity Score and Pain Interference Score, as well as an increase in Quality of Life Index were observed in CBD group than in control group, as assessed with CBPI questionnaire completed by the owners
Philpott *et al*., 2017 [72]	Animal study	Wistar rats	100–300 $\mu$ g CBD (i.a.) or vehicle	300 g$\mu$ CBD acted locally in the joint and reduced joint mechanical pain and inflammation, resulted in improvement in withdrawal threshold and weight bearing; No effect was shown at low dose CBD (100 and 200 µg)
Mejia *et al*., 2021 [73]	Prospective, randomized, double-blind, placebo-controlled, cross-over pilot animal study	Client-owned dogs with naturally occurring osteoarthritis of appendicular joints (n = 23)	2.5 mg/kg CBD (p.o.) or placebo every 12 h for 6 weeks	No significant changes in pain severity score between CBD and placebo group, assessed with objective gait analysis, activity counts and clinical metrology
Cunetti *et al*., 2018 [53]	Open-label study	Kidney transplant patients with chronic pain (fibromyalgia, osteoarticular, or neuropathic pain) who requested CBD for pain treatment (n = 7)	150 mg CBD (p.o.) twice daily for 3 weeks (titrated from 50 mg)	A decreased in pain score index and pain limitation perception were shown in 6 out of 7 patients, leading to partial or total pain improvements
Bebee *et al*., 2021 [74]	Randomized, double-blind, placebo-controlled study	Patients who presented with acute, non-traumatic low back pain (n = 100)	400 mg single-dose CBD (p.o.) or placebo	No significant reduction in pain score (CBD vs placebo: 6.2 vs 5.8 points) or hospital length of stay (CBD vs placebo: 9.0 vs 8.5 h) for people seeking help at a hospital emergency department for acute low back pain
Dieterle *et al*., 2022 [75]	Randomized, double-blind, placebo-controlled, cross-over study	Healthy participants (n = 24)	1600 mg single-dose CBD (p.o.) or placebo	No significant difference on opioid-induced hyperalgesia, allodynia, or pain between CBD and placebo group
*Parkinson's disease*				
Lastres-Becker *et al*., 2005 [78]	Animal study	Sprague–Dawley rats, injected with 6-hydroxydopamine	3 mg/kg CBD (i.p.) daily for 2 weeks	CBD showed an increase in the dopamine level in the brain
Chagas *et al*., 2014 [55]	Randomized, double-blind, placebo-controlled study	Subjects with Parkinson’s disease but without dementia or comorbid psychiatric conditions (n = 21)	75 or 300 mg CBD (p.o.) or placebo daily for 6 weeks	Improvement in daily living activities or well-being from 300 mg CBD in PDQ-39 but did not alter clinical motor symptoms as assessed with UPDRS
Leehey *et al*., 2020 [56]	Open-label study	Subjects with Parkinson’s disease (n = 13)	Epidiolex (titrated from 5 to 20–25 mg/kg) daily for 10–15 days	Improvements in total and motor functions of patients by 17.8% and 24.7%, respectively; Improvements in night-time sleep and emotional or behavioral dyscontrol scores by 49.1% and 10.6%, respectively, assessed with UPDRS
*Alzheimer's disease*				
Esposito *et al*., 2007 [82]	Animal study	C57BL/6 J mice, injected with Aβ	2.5 or 10 mg/kg CBD (i.p.) or vehicle (Tocrisolve 100) daily for 7 days	Dose-dependent inhibition of GFAP mRNA (-31.3 and -81%) and protein expression (-31 and -64.1%), as well as inhibition of iNOS (-33.3 and -61.5%) and IL-1β protein expression (-30.5 and -68%), and the related NO (-30 and -51%) and IL-1β release (-31 and -46.7%) in CBD group
Martin-Moreno *et al*., 2011 [57]	Animal study	C57BL/6 J mice, injected with Aβ	20 mg/kg CBD (i.p.) or vehicle daily for 1 week, then 3 days/week for 2 weeks	Prevention of both Aβ-induced cognitive impairment as tested with Morris water maze, and the increased gene expression of IL-6 but not TNF-α in CBD group
Cheng *et al*., 2014 [58]	Animal study	APP_swe_/PS1ΔE9 double transgenic C57BL/6 J × C3H/HeJ mice or wild type-like mice (n = 45)	20 mg/kg CBD (i.p.) or vehicle (control) daily for 3 weeks	Improvements in social recognition and object recognition memory in the CBD-treated transgenic mice, as assessed with SPT and NORT
*Huntington’s disease*				
Consroe *et al*., 1991 [84]	Randomized, double-blind, placebo-controlled, cross-over study	Subjects with Huntington’s disease (n = 15)	10 mg/kg CBD (p.o.) or placebo daily for 6 weeks	No significant difference in the improvement in chorea severity or other symptoms between CBD and placebo group
*Depression*				
Xu *et al*., 2019 [86]	Animal study	ICR mice (n = 48)	10 mg/kg CBD (i.v.) or 10 or 100 mg/kg CBD (p.o.) for 21 days	Low-dose IV and high-dose oral CBD showed a shorter mobility time (around 200 s) in forced swim test; Chronic use of CBD also appeared to reverse the depression-induced symptoms of chronic stress
Solowij *et al*., 2018 [59]	Open-label study	Frequent cannabis users (n = 20)	200 mg CBD (p.o.) daily for 10 weeks, while subjects continued usual usage of cannabis	Reduction in BDI score (BL 2.5 vs PT 0.5), improvement in RAVLT performance (Total words recalled from BL 52.55 to PT 56.00) and AST (Latency switching trials from BL 600.63 to PT 549.3 ms), showing an improvement in depressive and cognitive symptoms
Allsop *et al*., 2014 [87]	Randomized, double-blind, placebo-controlled study	Cannabis-dependent treatment seekers (n = 51)	Nabiximols (up to 86.4 mg THC and 80 mg CBD) or placebo for 6 days	Reduction in withdrawal-related depression (from BL 2.78 to abstinence 2.04) and other symptoms like sleep difficulty (from BL 2.95 to abstinence 2.79), restlessness (from BL 3.44 to abstinence 2.8), and decreased appetite (from BL 3.56 to abstinence 2.12) in treatment group
Berger *et al*., 2022 [60]	Open-label, single-arm phase II trial	Young people with anxiety disorders who did not respond to standard treatment with CBT and/or antidepressant (n = 31)	Up to 800 mg CBD (p.o.) daily for 12 weeks, in addition to standard treatment	Significant improvement in depressive symptoms, assessing using QIDS-A_17_ (BL 11.7 vs PT 8.2)
*Anxiety*				
Fogaca *et al*., 2018 [88]	Animal study	Chronically stressed C57BL/6 J mice and wistar rats	30 mg/kg CBD (i.p.) for 14 days	CBD prevented anxiogenic and depressogenic-like behaviors in EPM and NSF tests and displayed a neuroprotective effect
Resstel *et al*., 2009 [89]	Animal study	Wistar rats	1, 10 or 20 mg/kg single-dose CBD (i.p.) administered 30 min before exposure to restraint	Reduction of cardiovascular responses induced by restraint stress and attenuation of anxiogenic-like responses in EPM test
Guimarães *et al*., 1990 [91]	Animal study	Wistar rats	2.5, 5, 10 or 20 mg/kg single-dose CBD (i.p.) administered 60 min before EPM test	Significant increase in the entry ratio in EPM test at lower doses of CBD (2.5, 5 and 10 mg/kg) which showed anxiolytic-like effect of CBD; It also demonstrated inverted U shape dose–effect curve, with highest anxiolytic effect at 5 mg/kg
Zuardi *et al*., 1993 [90]	Randomized, double-blind, placebo-controlled study	Healthy subjects (n = 40)	300 mg CBD (p.o.), 10 mg diazepam, or 5 mg ipsapirone administered before SPS test	CBD reduced anxiety level after the SPS test, assessed using VAMS anxiety factor
Bergamaschi *et al*., 2011 [61]	Randomized, double-blind, placebo-controlled study	Treatment naïve subjects with generalized SAD (n = 24) and healthy control subjects (n = 12)	600 mg single-dose CBD (p.o.) or placebo administered 150 min before SPS test	CBD reduced symptoms of anxiety, cognitive impairment, and discomfort caused by public speech and decreased alertness during anticipatory speech in SAD subjects
Linares *et al*., 2019 [92]	Randomized, double-blind, placebo-controlled study	Healthy subjects (n = 57)	150, 300, 600 mg single-dose CBD (p.o.) or placebo	Significant anxiolytic effects in a SPS test with 300 mg CBD, assessed using VAMS anxiety factor; No significant differences in VAMS scores with 150 and 600 mg CBD
Zuardi *et al*., 2017 [93]	Randomized, double-blind, placebo-controlled study	Healthy subjects, with no history of past or current psychiatric illness, alcohol, or other drug dependence (n = 60)	100, 300, 900 mg single-dose CBD (p.o.), 1 mg clonazepam (p.o.) or placebo administered 150 min before SPS test	Dose-dependent anxiolytic effects in a SPS test assessed using VAMS anxiety factor, where anxiety levels reduced with 300 mg CBD, but not with 100 and 900 mg, in the post-speech phase
Berger *et al*., 2022 [60]	Open-label, single-arm phase II trial	Young people with anxiety disorders who did not respond to standard treatment with CBT and/or antidepressant (n = 31)	Up to 800 mg CBD (p.o.) daily for 12 weeks, in addition to standard treatment	Significant reduction in anxiety, assessing using OASIS (BL 10.8 vs PT 6.3) and HARS (BL 21.9 vs PT 11.3)
Masataka, 2019 [94]	Randomized, double-blind, placebo-controlled study	Japanese teenagers with social anxiety disorder and avoidant personality disorder (n = 37)	300 mg CBD (p.o.) or placebo daily for 4 weeks	A reduction in FNE (CBD vs placebo: -5.2 vs -0.2) and LSAS score (CBD vs placebo: -12.1 vs -3.1), showing a significant decrease in anxiety in CBD group
Hundal *et al*., 2018 [95]	Randomized, double-blind, placebo-controlled study	Non-clinical subjects with high paranoid traits (n = 32)	600 mg single-dose CBD (p.o.) or placebo, administered 130 min before entering virtual reality	Increased anxiety in BAI (BL 2.1 vs PT 6.8); No significant difference on persecutory ideation (CBD vs placebo: 13.4 vs 11.1 in SSPS) and physiological effects (cortisol and cardiovascular responses)
Arndt *et al*., 2017 [96]	Randomized, double-blind, placebo-controlled study	Healthy, drug-free subjects (n = 38)	300, 600 or 900 mg single-dose CBD (p.o.) or placebo	No significant reduction and difference on the responses to negative emotional stimuli and subjective effects between CBD and placebo group
*Psychosis*				
Hallak *et al*., 2010 [101]	Double-blind, placebo-controlled study	Subjects with schizophrenia (n = 28)	300 or 600 mg single-dose CBD (p.o.) or placebo administered 60 min before the assessment	No significant improvements on selective attention in both CBD groups in SCWT
Boggs *et al*., 2018 [100]	Randomized, double-blind, placebo-controlled parallel group, fixed-dose study	Subjects with chronic schizophrenia (n = 41)	600 mg CBD (p.o.) or placebo daily for 6 weeks, in addition to regular antipsychotic treatment	No significant improvement in psychotic symptoms and cognitive performance in CBD group, assessed using PANSS and MCCB scores respectively
McGuire *et al*., 2018 [62]	Randomized, double-blind, placebo-controlled parallel group study	Subjects with schizophrenia (n = 88)	1000 mg CBD (p.o.) or placebo daily for 6 weeks, in addition to regular antipsychotic treatment	Reduction in PANSS positive score by 1.4 in CBD group but no significant improvement in cognitive performance and overall functioning, assessed using BACS and GAF
Leweke *et al*., 2012 [28]	Phase 2, randomized, double-blind, parallel-controlled study	Subjects with schizophrenia and schizophreniform psychosis (n = 42)	Up to 800 mg CBD (p.o.) or 800 mg amisulpride (p.o.) daily for 4 weeks	Reduction in PANSS total score by 30.5 and 30.1 in CBD and amisulpride group respectively, showing similar efficacy of both in treating psychotic symptoms. CBD showed fewer extrapyramidal symptoms, less weight gain, and lower prolactin increase
Zuardi *et al*., 2009 [102]	Open-label pilot study	Subjects with Parkinson’s disease who had psychosis for at least 3 months (n = 6)	Flexible dose of CBD (p.o.) (initiated with 150 mg daily) for 4 weeks, in addition to usual therapy	Rapid onset of antipsychotic effects with an improvement in BPRS total score (from 18.5 to 5.5), PPQ total score (from 13 to 1.5), while not worsening symptoms of parkinsonism and cognitive function (UPDRS total score from 67.5 to 51.5; CGI-Improvement from 4 to 1.5)
*Substance use disorders*				
Ren *et al*., 2009 [105]	Animal study	Long-Evans rats	5 or 20 mg/kg CBD (i.p.)	Attenuation of cue-induced heroin seeking behavior. Protracted effect showed after 24 h and 2 weeks post-administration. No effects on heroin self-administration and extinction behavior
Parker *et al*., 2004 [108]	Animal study	Experimentally naive Sprague–Dawley rats	5 mg/kg CBD (i.p.) or vehicle administered prior to cocaine- and amphetamine-induced conditioned place preference	Less time spent on the cocaine- and amphetamine-paired floor, indicating that CBD potentiated the extinction of both cocaine- and amphetamine-induced conditioned place preference learning in CBD group
Viudez-Martinez *et al*., 2018 [115]	Animal study	C57BL/6 J mice	30, 60 or 120 mg/kg CBD (i.p.) daily	Reduction in ethanol consumption (from about 6 g of pure ethanol/kg body weight/day to 3.5 g/kg/day) and preference (from 75 to 55%), and hypothermia and handling-induced convulsion
Gonzalez-Cuevas *et al*., 2018 [116]	Animal study	Wistar rats with alcohol or cocaine self-administration histories (n = 24)	Transdermal CBD (2.5 g/100 g gel) or vehicle at 24 h intervals for 7 days	Attenuation of context-induced drug seeking for up to 5 months post-treatment, reduction in experimental anxiety and prevention in impulsivity associated with alcohol dependence in CBD group
Solowij *et al*., 2018 [59]	Open-label study	Frequent cannabis users (n = 20)	200 mg CBD (p.o.) daily for 10 weeks, while subjects continued usual usage of cannabis	Reduction in CEQ euphoria levels associated with cannabis use (BL 43.75 vs PT 38.65) but no significant changes in the frequency (BL 25 vs PT 30 days per month) and quantity of cannabis use (BL 123.75 vs PT 105.0 cones)
Hurd *et al*., 2019 [106]	Randomized, double-blind, placebo-controlled pilot study	Subjects with opioid dependence, abstinent for at least 7 days (n = 42)	400, 800 mg CBD (p.o.) or placebo daily for 3 consecutive days	Reduction in the cue-induced heroin craving (mean difference in VAS-C score for placebo vs 400 mg vs 800 mg: 0.93 vs 0.44 vs 0.23) and anxiety (mean difference in VAS-A score for placebo vs 400 mg vs 800 mg: 0.97 vs 0.48 vs 0.24). Protracted effect showed after 24 h and 7 days post-administration
Mongeau-Perusse *et al*., 2021 [110]	Phase 2, randomized, double-blind, placebo-controlled parallel-group study	Subjects with moderate-to-severe cocaine use disorder (n = 78)	800 mg CBD (p.o.) or placebo daily for 12 weeks	No reduction in cocaine-cue-induced craving and prevention in relapse, as seen from an increased craving scores (CBD vs placebo: 4.69 vs 3.21), and a shorter times-to-cocaine relapse (CBD vs placebo: 4 vs 7 days)
Morgan *et al*., 2013 [112]	Randomized, double-blind, placebo-controlled study	Regular smokers (> 10 cigarettes/day) who wished to stop smoking (n = 24)	400 μg of inhaled CBD or placebo for 7 days, administered when subjects felt the urge to smoke	Reduction in cigarette consumption (pre-treatment 90 vs PT 50 cigarettes/week) without increased in nicotine craving (TCQ Day 0 43.83 vs Day 7 37.08) in CBD group
Hindocha *et al*., 2018 [113]	Randomized, double-blind, placebo-controlled, cross-over study	Non-treatment seeking, nicotine-dependent cigarette smokers (n = 30)	800 mg single-dose CBD (p.o.) or placebo, followed by overnight abstinent sessions	Reduction in salience and pleasantness of cigarette cues (valence bias of CBD vs placebo: 0.59 vs 1.10) in CBD group; No significant effects on tobacco craving or symptoms of withdrawal
Karoly *et al*., 2021 [117]	Observational study	Cannabis and alcohol using adults (n = 120)	One of the three cannabis products (THC-dominant, CBD-dominant or CBD + THC) administered for 5 days	Reduction in quantity and frequency of alcohol consumed in CBD group (Percent alcohol use days: CBD vs THC + THC/CBD = 25% vs 34%)
Haney *et al*., 2016 [119]	Randomized, placebo-controlled, cross-over study	Non-treatment seeking, healthy cannabis smokers (n = 31)	200, 400, 800 mg single-dose CBD (p.o.), or the placebo administered 90 min prior to cannabis administration	No significant reduction in reinforcing (self-administration), physiological (e.g. heart rate), or positive subjective effects (e.g. 'high', 'good effect', 'liking') of smoked cannabis at any doses of CBD
Freeman *et al*., 2020 [120]	Phase 2a, randomized, double-blind, placebo-controlled study	Subjects with cannabis use disorder (n = 48)	200, 400 or 800 mg CBD (p.o.) or the placebo for 4 weeks during a cessation attempt	400 and 800 mg CBD decreased THC-COOH:creatinine ratio by 94.21 and 72.02 ng/mL, respectively and increased abstinence from cannabis by 0.48 and 0.27 days per week, respectively
*Insomnia*				
Monti, 1977 [63]	Animal study	Wistar rats (n = 8)	20 or 40 mg/kg single-dose CBD (i.p.)	Reduction in slow-wave sleep latency by 16 and 13 min at 20 and 40 mg/kg, respectively. Reduction in wakefulness time and increase in slow-wave sleep time by 39 min at 40 mg/kg
Carlini, & Cunha, 1981 [64]	Randomized, double-blind, placebo-controlled, cross-over study	Subjects with insomnia (n = 15)	Single dose of 40, 80, 160 mg CBD (p.o.), nitrazepam 5 mg (p.o.), or placebo administered 30 min before bed	10 out of 15 subjects experienced longer sleep at 160 mg CBD; Less participants recall their dreams (5–6 out of 15 subjects) at all doses of CBD
Shannon *et al*., 2019 [122]	Retrospective case series	Subjects with sleep (n = 25) or anxiety disorder (n = 47)	25–175 mg CBD (p.o.) daily for 3 months	79.2% and 66.7% of patients showed improvement in anxiety and sleep, respectively, in the first month
*Weight or appetite*				
Ignatowska-Jankowska *et al*., 2011 [123]	Animal study	Wistar rats (n = 114)	2.5, 5 mg/kg CBD (i.p.), saline or vehicle daily for 14 days	CBD showed a reduction in body weight gain in rats (weight gain in saline/ vehicle vs CBD at day 14 of treatment: 40 vs 30 g)

Abbreviations: APP, Amyloid precursor protein; AST, Attentional Switching Task; Aβ, β-amyloid; BACS, Brief Assessment of Cognition in Schizophrenia; BAI, Beck Anxiety Inventory; BDI, Beck Depression Inventory; BL, baseline; BPRS, Brief Psychiatric Rating Scale; CBPI, Canine Brief Pain Inventory; CBT, Cognitive behavioral therapy; CEQ, Cannabis Experiences Questionnaire; CGIC, Caregiver Global Impression of Change; CGI, Clinical Global Impression scale; EPM, elevated plus maze; FNE, Fear of Negative Evaluation Questionnaire; GAF, Global Assessment of Functioning; GFAP, Glial fibrillary acidic proteins; HARS, Hamilton Anxiety Rating Scale; i.a., intraarticular injection; i.p., intraperitoneal injection; i.v., intravenous injection; IL, Interleukin; iNOS; inducible Nitric Oxide Synthase; LSAS, Liebowitz Social Anxiety Scale; MCCB, MATRICS Consensus Cognitive Battery; NORT, Novel object recognition test; NSF, Novelty suppressed feeding; OASIS, Overall Anxiety Severity and Impairment Scale; p.o., oral administration; PANSS, Positive and Negative Syndrome Scale; PDQ, Parkinson’s Disease Questionnaire; PPQ, Parkinson Psychosis Questionnaire; PS1, Presenilin 1; PT, post-treatment; QIDS-A_17_, Quick Inventory of Depressive Symptoms; RAVLT, Rey Auditory Verbal Learning Test; S/CGIC, Subject/ Caregiver Global Impression of Change; SAD, Social Anxiety Disorder; SCWT, Stroop Color Word; SPSS, State Social Paranoia Scale; SPS, simulated public speaking; SPT, Social preference test; TCQ, Tiffany Craving Scale; TNF-α, Tumor necrosis factor-α; UPDRS, Unified Parkinson’s Disease Rating Scale; VAMS, Visual Analogue Mood Scale; VAS-A, Visual Analogue Scale for Anxiety; VAS-C, Visual Analogue Scale for Craving

#### The Effect of CBD on Neurological Conditions

##### Epilepsy

Epileptic patients often require one or more anticonvulsants to prevent recurrent seizures [65]. However, around 30% of patients remain resistant to treatment and do not respond to conventional anti-seizure medications [65]. CBD is now approved to treat drug-resistant epilepsies by various drug regulatory bodies around the world including in the US, Europe, and Australia [11, 66]. These approvals were supported by positive randomized controlled trials (RCTs) showing CBD reduced seizures in intractable epilepsies including Dravet syndrome, Lennox-Gastaut syndromes, and tuberous sclerosis complex (TSC) [3, 52, 67].

Two clinical trials were conducted on patients with Dravet and Lennox-Gastaut syndromes [3, 52]. Oral CBD (10 or 20 mg/kg/day) was administered in conjunction with one or more of their standard antiepileptic treatments (clobazam, valproates, lamotrigine, and/or levetiracetam) for 14 weeks. CBD was found to reduce the frequency of convulsive and drop seizures by 37–42%, as compared to less than 17.2% reduction in the placebo group. The overall condition improved in more than 50% of patients [3, 52]. Additionally, two open-label extension studies supported that the long-term use of 20–30 mg/kg/day oral CBD decreased the frequency of seizures by 45–84% through 156 weeks of treatment [68, 69]. The majority of patients (≥ 83%) also experienced positive changes in their overall condition [68, 69].

The use of CBD in refractory seizures associated with TSC was studied by Hess et al. [4]. Patients with TSC associated with drug-resistant epilepsy were given a maximum of 50 mg/kg/day CBD for 3 months. A significant reduction by nearly half (48.8%) in weekly seizure frequency was recorded when compared to baseline [4]. Thiele et al. conducted a similar clinical trial in patients with TSC showed that 16-week CBD administration (25 or 50 mg/kg/day) reduced seizure frequency by 47–49%, which was about 20% more reduction than the placebo group (26.5%) [67]. Subsequently, Thiele et al. extended the study in these patients and demonstrated that a mean modal dose of 27 mg/kg/day resulted in a 54–68% reduction in seizure frequency through 48 weeks of treatment, with 53–61% and 87% of patients experiencing at least 50% reduction in seizures and improvement in overall condition, respectively [70].

##### Pain

CBD has been reported to have analgesic effects, especially in chronic non-cancer pain related to inflammatory conditions such as osteoarthritis [71]. A few animal studies were conducted to evaluate the effect of CBD on osteoarthritic pain. Rats receiving 300 µg intraarticular CBD injection showed a local action in the joint and a reduction in joint mechanical pain and inflammation, resulting in improvement in withdrawal threshold and weight-bearing tolerance [72]. Another study in dogs showed that a 12-week twice daily dosing of 2 mg/kg oral transmucosal CBD oil lowered the pain intensity and impact of osteoarthritis on daily activity [54]. Thus, the dogs experienced an increment in comfort and activity and an improvement in quality of life [54]. However, a similar pilot study that was also conducted in dogs with osteoarthritis pain over a shorter period (6 weeks) did not demonstrate the same positive result [73]. No significant improvement in pain severity score was observed after the administration of oral CBD oil (2.5 mg/kg) every 12 h [73].

Two clinical trials were done to evaluate the effect of single-dose CBD on people with acute low back pain, and on healthy participants with induced hyperalgesia, allodynia, and pain [74, 75]. A single oral dose of CBD (400 or 1600 mg) was administered in both trials, but CBD was not effective in reducing pain [74, 75]. Daily CBD dosing, however, was shown to improve pain in an open-label study [53]. Seven kidney transplant patients with different kinds of chronic pain (fibromyalgia, osteoarticular, neuropathic) were given oral CBD for three weeks, titrating from 100 to 300 mg/day. Partial or total pain improvements were experienced in six patients with a reduction in pain intensity and pain limitation perception [53]. Whilst there is promising evidence for the use of CBD in inflammatory pain conditions, systematic reviews have shown that oral and inhaled cannabis could only provide limited and temporary relief in chronic pain [76, 77]. However, none of these reviews assessed the effect of pure CBD due to the lack of standardized formulation used in previous RCTs. Larger scale and long-term placebo-controlled RCTs are needed to provide more definitive evidence on the use of CBD as an analgesic.

##### Parkinson’s Disease

Parkinson’s disease is a neurodegenerative disease characterized by movement disorder and non-motor dysfunction, which required multiple medications to control the symptoms [56]. CBD was previously tested in animal models of Parkinson’s disease and showed possible neuroprotective and antioxidant effects [78, 79]. In the experiment, 6-hydroxyldopamine was injected into rats, inducing dopamine depletion which mimicked the pathophysiology of Parkinson’s disease. Two-week treatment with CBD (3 mg/kg) increased dopamine concentrations in the brain [78].

Considering the promising result from animal studies, clinical trials were conducted. An open-label trial reported a reduction in disease severity (17.8%) and movement disorders (24.7%) after administering CBD (20–25 mg/kg/day) for 10–15 days in Parkinson’s disease patients [56]. Participants also experienced improvements in non-motor functions, for example in night-time sleep (49.1%) and emotional or behavioral dyscontrol (e.g. apathy, agitation, irritability, and impulsivity) (10.6%) [56]. However, mixed results were observed in a double-blind study of Parkinson’s disease patients [55]. A 6-week oral CBD (300 mg/day) administration improved daily living activities (i.e. washing, dressing, buttoning or tying shoe laces, writing clearly, cutting food, and holding a drink without spilling) but did not alter clinical symptoms when compared with placebo [55]. Further larger scale placebo-controlled clinical trials assessing the efficacy of CBD in Parkinson’s disease patients are required.

##### Alzheimer’s Disease

Alzheimer’s disease is another progressive neurodegenerative disease caused by the aggregation of β-amyloid (Aβ) plaques and neurofibrillary tangles, contributing to a decline in cognition [80]. Currently available drugs are symptom-modifying but not disease-modifying [80, 81]. Although there have been no human studies to date, the neuroprotective, antioxidant, and anti-inflammatory effects of CBD have been shown to reduce the pathological symptoms in multiple Alzheimer’s disease rodent models and have the potential to delay disease onset and progression [57, 82].

The mice in the studies conducted by Esposito et al. and Martin-Moreno et al. received Aβ intrahippocampal or intraventricular injections to simulate the pathophysiology of Alzheimer’s disease [57, 82]. CBD was then injected intraperitoneally (2.5, 10, or 20 mg/kg i.p.) and showed an improvement in cognitive performance. It also demonstrated a dose-dependent inhibition of glial fibrillary acidic protein expression, as well as a reduction in nitric oxide and various pro-inflammatory cytokines that are commonly elevated in Alzheimer’s disease (IL-1β and IL-6) [57, 82]. Unlike the previous animal studies, Cheng et al. mutated two genes (amyloid precursor protein and presenilin 1) that were known to be involved in the pathophysiology of Alzheimer’s disease in the mouse model [58]. Intraperitoneal CBD-treated transgenic mice showed improvements in social recognition and object recognition memory [58].

##### Huntington’s Disease

CBD was suggested to be a potential treatment for Huntington’s disease, an inherited neurodegenerative disease that mainly affects movement [83]. A small clinical study conducted by Consroe et al. reported CBD administered orally at 10 mg/kg oral CBD failed to improve chorea or other symptoms in Huntington’s disease patients [84]. The lack of activity observed could be due to the low oral bioavailability and low oral dose, which led to low CBD plasma levels (5.9–11.2 ng/mL) [84]. Further investigation is necessary with careful considerations in the study design, particularly on the selection of the dose, and the route of delivery.

#### The Effect of CBD on Psychiatric Conditions

##### Depression

Antidepressant effects of CBD have been examined mostly in animals [85]. Xu et al. conducted forced swim tests in the chronic mild stress mouse model and administered long-term periodic CBD via two routes [86]. Results demonstrated that low-dose intravenous (IV) and high-dose oral CBD exerted antidepressant-like effects in reducing immobility time [86]. The low-dose oral formulation, however, did not improve depression-related behavior, possibly due to poor oral bioavailability. It was also reported that persistent use of CBD could reverse depression-induced symptoms of chronic stress [86], supporting long-term CBD administration for its sustained antidepression effects.

Depressive disorders are usually associated with other illnesses, such as substance use disorder and anxiety. It is also commonly studied as a secondary outcome to the comorbid medical conditions. Solowij et al. showed that prolonged administration of CBD (200 mg/day for 10 weeks) in frequent cannabis users reduced depression-like symptoms and improved cognitive symptoms in terms of attentional switching, verbal learning, and memory [59]. Allsop et al. also conducted a randomized controlled trial on cannabis-dependent users [87]. However, nabiximols (a mixture containing THC and CBD) was used as an agonist replacement therapy in cannabis withdrawal. Nabiximols significantly reduced withdrawal-related depression and other symptoms, such as sleep disturbance, loss of appetite, and restlessness [87]. These findings indicated the potential use of CBD for depression but more studies are required, using only CBD as the treatment group and a larger sample size, to confirm the antidepression efficacy of CBD. A recent open-label study was conducted on young people with anxiety who were unresponsive to standard treatment, including cognitive behavioral therapy and/or antidepressants [60]. Coadministration of oral CBD (up to 800 mg/day) significantly reduced comorbid depressive symptoms severity by 29.9% 12 weeks post-treatment [60].

##### Anxiety

Anxiolytic effects of CBD have been investigated in various animal and human models [8, 85]. Multiple rat models demonstrated that CBD at different doses (1–30 mg/kg i.p.) prevented the anxiogenic response and related cardiovascular response in stress-induced rats [88, 89]. Simulated public speaking tests are often used to induce anxiety and its physiological response (e.g. increase in cortisol level, blood pressure, and heart rate) in healthy volunteers and a single-dose CBD was given a few hours prior to the test to determine its anxiolytic effects. Zuardi et al. and Bergamaschi et al. reported that oral CBD (300 or 600 mg) reduced the symptoms and level of anxiety in a stressful situation in both healthy participants and patients with generalized social anxiety disorder [61, 90]. It also reduced cognitive impairment, discomfort, and arousal associated with public speaking [61, 90]. The anxiolytic response was dose-proportional and followed an inverted U-shaped curve in some animal and human studies, in which effects were apparent at intermediate doses but not at low or high doses [91–93]. Therefore, selecting an appropriate dose is important as it may significantly affect efficacy.

Previously, most studies were performed on healthy participants with experimental anxiety induced by stress or simulated public speaking tests. Therefore, two recent trials were performed on patients who were clinically diagnosed with anxiety to explore the anxiolytic effects of CBD [60, 94]. In young patients with social anxiety disorder, a 4-week oral CBD (300 mg/day) treatment significantly reduced anxiety level when compared to the placebo [94]. An add-on CBD (up to 800 mg/day for 12 weeks) therapy also improved anxiety symptoms in patients who had anxiety that failed to respond to conventional treatment [60].

Despite the positive findings on the effects of CBD on anxiety, minimal psychological or behavioral effects were observed in several studies [95, 96]. Hundal et al. reported that pre-treatment with a single oral CBD dose (600 mg) appeared to increase anxiety in healthy volunteers with high paranoid traits after a virtual-reality session [95]. The anxiolytic properties of CBD remained inconsistent, and the variation in the outcome measures could be attributed to differences in the experimental setting, anxiety-induced stimulus, and tools for psychological measurements between the studies. While single dosing was used in most studies, the impact of daily CBD dosing regimen should be investigated in the future to determine the effectiveness of long-term administration on anxiety.

##### Psychosis

Psychotic disorders such as schizophrenia are characterized by positive symptoms (hallucinations and delusions), negative symptoms (blunted affect), and cognitive impairment (deficits in working memory) [97]. Psychosis is managed by first- and second-generation antipsychotic drugs, however, these pharmacotherapies have a myriad of undesirable effects such as extrapyramidal effects, hyperprolactinemia, weight gain, and metabolic issues [98]. There is a need to develop new generation antipsychotics that are better tolerated by patients. The efficacy and safety of CBD have been tested on schizophrenia patients [99].

Boggs et al. conducted a randomized controlled trial in antipsychotic-treated patients with chronic schizophrenia [100]. Administration of oral CBD (600 mg/day for 6 weeks) did not improve cognition and psychotic symptoms [100]. In contrast, similar clinical studies were performed by McGuire et al. but with a higher dose (1000 mg/day for 6 weeks) in patients with acute schizophrenia, resulting in improved schizophrenic symptoms when compared to placebo [28, 62]. The findings from these two studies suggest that 600 mg of CBD may not be adequate to significantly improve psychotic symptoms and CBD may be more effective in treating acute rather than chronic schizophrenia. Furthermore, Leweke et al. demonstrated comparable efficacy between CBD (800 mg/day) and a potent antipsychotic, amisulpride (800 mg/day), in reducing positive and negative schizophrenia symptoms [28]. CBD also showed better tolerability and safety than amisulpride, with fewer extrapyramidal symptoms, less weight gain, and lower prolactin increase [28]. Thus CBD was more favorable than conventional antipsychotic treatment. One of the major limitations in current antipsychotic drugs is their inability to treat cognitive impairment in patients. An early clinical study by Hallak et al. showed that a single dose of oral CBD (300 or 600 mg) did not significantly improve cognitive performance in schizophrenia [101]. Future studies are needed with higher doses and with repeated dosing regimens.

Psychosis is one of the common complications of Parkinson’s disease as anti-Parkinson medications increase dopamine concentrations in the brain [102]. Low dose antipsychotics are often used to treat psychosis in Parkinson’s disease, however, they risk worsening parkinsonism. CBD use in psychosis in Parkinson’s disease was analyzed by Zuardi et al. in a 4-week open-label pilot study [102]. CBD demonstrated a rapid antipsychotic effect without exacerbating parkinsonism symptoms and cognitive function [102].

### CBD on Substance Use Disorders

Substance use disorders can be characterized by drug addiction and a person's inability to control their use of drugs of misuse [103]. Research on animals and humans was performed to explore the potential use of CBD in reducing cravings and substance use related to opioids, psychostimulants, tobacco, alcohol, and cannabis. Its ability in preventing relapse and reducing symptoms associated with substance withdrawal such as anxiety and hypothermia was also investigated [103].

#### Opioid-Related Addictive Behaviors

Opioid-related addiction and misuse have been a major health issue for years [104]. Limited treatments for opioid use disorder are available nowadays, one of which is opioid agonist therapy using methadone and buprenorphine [104]. However, the uptake of therapy has remained low due to poor accessibility and stigma [104] so there is a need for new therapeutic strategies. CBD has recently been proposed as a potential treatment for opioid use disorder. A preclinical study using a self-administration rat model showed that CBD (5 or 20 mg/kg i.p.) attenuated conditioned cue-induced heroin seeking behavior for up to 14 days post-administration [105]. Subsequently, Hurd et al. replicated the experimental design to determine whether similar effects could be observed in humans [106]. Oral CBD (400 or 800 mg/day) or placebo was given for three consecutive days to drug-abstinent adults with heroin use disorder [106]. CBD reduced cue-induced craving and anxiety for up to 7 days post-treatment [106].

The protracted effects of CBD would potentially be beneficial clinically, particularly for patients with poor adherence to their medications. Additionally, both studies above investigated the effects of CBD on heroin addiction, yet the rise in prescription opioid use also contributes crucially to opioid crisis [104]. Future studies should explore the effects of CBD on prescription opioids as well, such as codeine, oxycodone, tramadol, and fentanyl.

#### Psychostimulant-Related Addictive Behaviors

Cocaine is one of the frequently misused illicit drugs associated with severe health and social problems [107]. There is no efficacious treatment currently available for cocaine use disorder that reduces craving and prevents relapse [107]. With promising findings of CBD for addiction of other substances, research interest has increased in examining its effect on psychostimulant addiction. Preclinical studies have been performed by injecting CBD (5 mg/kg i.p.) into rats after cocaine- and amphetamine-induced conditioned place preference [108]. An extinction trial was then given and found that CBD-treated mice spent less time on the psychostimulants-paired floor as compared with vehicle-treated mice, meaning that CBD potentiated the extinction of place preference learning induced by both psychostimulants [108]. CBD was also found to be protective against seizures and acute liver injury associated with cocaine intake in mice [109].

However, conflicting results were observed in the clinical study by Mongeau-Pérusse et al. [110]. Daily oral CBD (800 mg) was administered in adults with cocaine use disorder for 12 weeks [110]. The higher increase in craving scores (CBD vs placebo: 4.69 vs 3.21) and shorter median times-to-cocaine relapse (CBD vs placebo: 4 vs 7 days) suggested that CBD did not reduce cocaine craving and prevent relapse [110]. Subjective measures of relapse and urine test dates known by the patient were utilized in this study, which could lead to bias and unreliability in the results [110]. Further research with objective outcome measurements is needed to determine whether CBD has the potential to reduce psychostimulant addiction.

#### Tobacco-Related Addictive Behaviors

Nicotine dependence is a common issue in cigarette smokers due to the euphoria from nicotine. The withdrawal effects make smoking cessation difficult and increase the risk of relapse [111]. Currently, different forms of nicotine replacement therapy are used for smoking cessation [111]. CBD has also been tested to explore its effect on nicotine addiction, maintaining long-term abstinence, and preventing relapse.

Morgan et al. conducted a randomized controlled trial on smokers who intended to quit smoking [112]. They were given CBD (400 µg/dose) via pressurized metered dose inhalers (MDIs) or a placebo inhaler and were told to inhale when they experience nicotine cravings. The number of cigarettes smoked and usage of the inhaler were recorded [112]. The CBD inhalers reduced cigarette smoking by 40 cigarettes per week, compared to a decrease of only 10 cigarettes per week in the placebo group [112]. Craving intensity did not increase during nicotine withdrawal. In fact, a decrease in the craving score was observed [112]. Subsequently, a cross-over study was done by Hindocha et al. [113]. A single oral dose of CBD (800 mg) or placebo was given during overnight smoking cessation. CBD was shown to reduce attentional bias and pleasantness of cigarette cues but there was no effect on nicotine craving and withdrawal [113]. Larger and longer studies are required to conclude the effect of CBD on tobacco-related addictive behaviors.

#### Alcohol-Related Addictive Behaviors

Alcohol use disorder has been associated with a wide range of long-term health issues, including heart, liver, and gastrointestinal problems [114]. Excessive alcohol intake can also result in risky behaviors [114]. Relapse rate is still high, despite receiving detoxification. Therefore, new treatments have always been sought to help reduce the risk of relapse, craving, and withdrawal symptoms. A preclinical study in mice showed that CBD (30, 60, and 120 mg/kg/day i.p.) reduced ethanol intake (from about 6 to 3.5 g/kg/day) and preference (from 75 to 55%), as well as some of the negative impacts caused by alcohol consumption and withdrawal, e.g. hypothermia and handling-induced seizures [115]. Transdermal CBD was utilized to produce sustained plasma CBD levels in another preclinical study [116]. Application of 2.5 g CBD/100 g gel on mice reduced the number of responses during alcohol-induced reinstatement and maintained for up to 5 months post-treatment. A reduction in withdrawal symptoms, including anxiety and impulsivity, could also potentially help prevent relapse [116]. Karoly et al. conducted an observational study on cannabis and alcohol users following the promising findings of CBD on alcohol addiction from animal studies [117]. It showed that the CBD-predominant treatment (24% CBD and 1% THC) reduced alcohol consumption when compared with the THC-predominant group (24% THC and 1% CBD) and the CBD + THC group (10% CBD and 9% THC) [117].

#### Cannabis-Related Addictive Behaviors

THC is one of the psychoactive ingredients that contributes to cannabis addiction and intoxication [118]. CBD has been explored in its ability to reverse the effects of THC and to reduce craving and prevent relapse. A 10-week open-label trial by Solowij et al. found that CBD (200 mg/day) decreased euphoria associated with cannabis use, which may lower the incentive to continue using cannabis [59]. However, the study showed no significant changes in cannabis frequency and quantity used in frequent cannabis users [59]. Haney et al. performed a clinical trial on non-treatment-seeking healthy cannabis smokers, and a single-dose oral CBD (0, 200, 400, or 800 mg) pre-treatment reported no significant effect on cannabis self-administration, euphoria, other reinforcing effects, as well as cardiovascular effects associated with cannabis use [119]. Freeman et al. used the same CBD doses but for a longer duration of 4 weeks [120]. They showed that 400 and 800 mg CBD, but not 200 mg, increased self-reported abstinence from cannabis by half a day per week when compared with placebo [120]. The difference in the duration of treatment may explain the variation in the treatment outcome. Therefore, future research is essential to confirm the efficacy of CBD on cannabis use disorder.

#### CBD on Other Neuropsychiatric Conditions

##### Insomnia

Insomnia is a common sleep disorder associated with many mental conditions, including depression, anxiety, and psychosis [121]. People with insomnia often have a shorter duration of sleep, decreased slow-wave sleep time, and increased wakefulness time [63]. Currently available pharmacological treatments include first-generation antihistamines, benzodiazepines, antidepressants, and antipsychotics, which have undesirable adverse effects such as tolerance, dependence, and over-sedation [121]. The hypnotic and sedative effects of CBD have been investigated for its ability to regulate the sleep–wake cycle to improve sleep for patients with insomnia.

Monti examined the effects of acute CBD administration on sleep in rats [63]. Acute exposure to CBD (20 or 40 mg/kg i.p.) reduced the time to reach slow-wave sleep. A higher dose (40 mg/kg CBD) also reduced the time spent awake and increased slow-wave sleep time [63]. Later, Carlini and Cunha found that two-thirds of insomniacs experienced a significant increase in sleep duration (more than 7 h) with 160 mg oral CBD as compared with placebo, a lower dose of CBD (40 and 80 mg), and 5 mg nitrazepam [64]. Additionally, about two-thirds of participants reported less dream recall, which could potentially reduce the number of times waking up at night. They also indicated that CBD was less likely to cause over-sedation or concentration difficulty the next morning, which would be better than conventional treatment [64]. A retrospective case series analyzed the effectiveness of CBD for treating sleep disturbances [122]. An improvement in sleep was reported by 66.7% of patients, while 25% experienced worsening of symptoms in sleep, and a sustained response was not observed throughout the 3-month study [122]. Further analysis is needed to fill the knowledge gap in the efficacy of CBD on long-term sleep quality.

##### CBD on Weight or Appetite

CBD has been thought to play important roles in energy balance, food intake, and body weight control. A 2-week CBD treatment (2.5 or 5 mg/kg i.p.) decreased the body weight gain by 10 g compared to vehicle-treated rats [123]. Decreased appetite had also been reported as a side effect in some human studies after oral CBD administration [3, 4, 52, 67]. However, an increased appetite or hunger was found in CBD-treated groups in some other studies [71, 95, 124]. Since current evidence is scarce and inconclusive, more research is required to understand its effects on body metabolism, appetite, or weight-related body functions. The potential use of CBD as an appetite stimulant could be useful in patients with HIV infection, cancer-related anorexia, chronic pain, or mental illnesses. On the other hand, CBD as an appetite suppressant may benefit patients with conditions that require weight loss, for example, obesity, diabetes, and cardiovascular diseases.

To date, most clinical research examined the use of CBD in epilepsy, which is the only approved indication that has gone through Phase III trials. On the other hand, current evidence for other brain disorders is still in the early phases. Despite this, CBD has shown high potential in treating these conditions, but more research is needed for conclusive clinical data.

## Pharmacokinetics of CBD according to Different Routes of Administration

The pharmacological effects and PK of CBD can vary depending on the route of administration (intravenous, oral, sublingual, pulmonary, intranasal, and transdermal) (Table III) and are impacted by the physicochemical properties of CBD. Absorption is impaired by its high lipophilicity (*log P* = 6.3) and low water solubility (12.6 mg/L) [14]. CBD has a high volume of distribution (32.7 L/kg) [125, 126], reflecting wide distribution into different tissues. Following systemic absorption, one of the major targets for CBD is the brain, allowing the potential treatment for CNS diseases. For most administration routes, CBD needs to cross the blood–brain barrier to achieve blood-to-brain delivery. Due to its lipophilic nature, CBD can readily cross the blood–brain barrier via passive diffusion [127]. The brain disposition of CBD does not appear to be influenced by active transport and more research is needed in this area. A knockout mice study reported that the brain uptake of CBD was not influenced by murine forms of P-glycoprotein (P-gp) and breast cancer resistance protein (BCRP) [128, 129]. A more recent study reported that CBD was not a P-gp substrate and that CBD was a weak BCRP substrate based on Transwell assays using Madin-Darby Canine Kidney II (MDCK) cells overexpressing human transporters [129]. CBD is highly protein bound (86.7–92.2%) [130], which may influence its distribution to the brain and its activity in CNS disorders. The metabolism and excretion of CBD can also be altered as it undergoes significant first pass metabolism by various cytochrome P450 (CYP450) isozymes in the liver, primarily CYP2C19 and CYP3A4 [131]. When CBD is taken orally, it is metabolized into 7-hydroxyl-CBD and further inactivated into 7-carboxy-CBD, which has a 40-fold higher blood concentration than CBD [15]. After CBD dosing, the reported elimination half-life ranges from 1 h to 5 days, depending on the dose and route of administration, as well as the monitoring duration of the studies [13].

**Table III:** Studies Reporting Plasma PK Parameters for CBD

References	Subjects	Administration route	Dose (mg)	Plasma PK parameter							
				t_max_ (h)	C_max_ (ng/mL)	t_1/2_ (h)	AUC (ng·h/mL)	CL (L/h)	Vd (L)	MRT (h)	F (%)
Xu *et al*., 2019 [86]	Mouse	Oral	20 mg/kg	2	129.5	ND	551	51.2 L/h/kg	215.3 L/kg	4.2	8.63
		IV	10 mg/kg	0.167	2343.3	3.9	3191	3.4 L/h/kg	19.5 L/kg	3.3	ND
Deiana *et al*., 2012 [138]	Mouse	Oral (in plasma)	120 mg/kg	1	2.2 µg/mL	ND	378 µg·min/mL	ND	ND	ND	ND
		Oral (in brain)	120 mg/kg	6	1.3 µg/mL	ND	319 µg·min/mL	ND	ND	ND	ND
	Rats	Oral (in plasma)	120 mg/kg	2	2 µg/mL	4.62	536 µg·min/mL	ND	ND	ND	ND
		Oral (in brain)	120 mg/kg	4	8.6 µg/mL	3.7	1900 µg·min/mL	ND	ND	ND	ND
Paudel *et al*., 2010 [142]	Rats	Intranasal	200 µg/kg	0.48	27.35	1.31	43.26	4.83 L/h/kg	ND	ND	34–46
		IV	200 µg/kg	ND	3596	1.06	103.56	10.21 L/h/kg	7.59 L/kg	ND	ND
	Guinea pigs	Transdermal gel	18 mg/mL	38.4	8.6	ND	276	ND	ND	ND	ND
		IV	1 mg/kg	ND	269	3.47	175	3.22 L/h/kg	13.72 L/kg	ND	ND
Spittle *et al*., 2021 [140]	Guinea pigs	Oral	25 mg/kg	1.6	42	8.1	379.5	77.7 L/h/kg	693.5 L/kg	8.6	ND
			50 mg/kg	4.8	96.8	10.8	873.7	63.8 L/h/kg	685.9 L/kg	11.3	ND
Meyer *et al*., 2022 [136]	Dairy calves	Oral	5 mg/kg	7.5	0.05 µg/mL	23.02	0.7 µg·h/mL	5.29 L/h/kg	175.86 L/kg	35	ND
Yocom *et al*., 2022 [139]	Horses	Oral	1 mg/kg	4.1	4.3	14.8	51.6	8265.7	216.7	13.5	ND
			3 mg/kg	5	19.9	8.5	162.9	10,881.3	214.2	10.7	ND
Williams *et al*., 2022 [134]	Horses	Oral	0.35 mg/kg	1.8	6.6	ND	42	ND	170	156	ND
			2 mg/kg	2.4	51	13.3	330	ND	131	153	ND
Samara *et al*., 1988 [133]	Dogs	IV	45	ND	ND	6.8	2706 µg·h/L	17.3	167	ND	ND
			90	ND	ND	9.3	6095 µg·h/L	15.9	209	ND	ND
		Oral	180	ND	ND	ND	ND	ND	ND	ND	13–19
Polidoro *et al*., 2022 [157]	Dogs	Intranasal	20	0.5	28	ND	61	ND	ND	ND	ND
		Oral	100	3.5	217	ND	1376	ND	ND	ND	ND
Bartner *et al*., 2018 [165]	Dogs	Oral	75	ND	625.3	3.33	2.26	ND	ND	3.6	ND
			150	ND	845.5	2.13	4.96	ND	ND	5.0	ND
		Transdermal cream	75	ND	74.3	ND	0.20	ND	ND	8.2	ND
			150	ND	277.6	ND	0.50	ND	ND	7.8	ND
Taylor *et al*., 2018 [137]	Human	Oral	1500	4	292.4	ND	1517	1111	20,963	1517	ND
			3000	5	533	ND	2669	1121	23,357	2669	ND
			4500	5	722.1	ND	3215	1445	36,575	3215	ND
			6000	5	782	ND	3696	1909	42,849	3696	ND
Vitetta *et al*., 2021 [144]	Human	Oro-buccal	6	1	0.53	1.23	0.9	6900	ND	ND	ND
			18	1	4.62	5.45	8.9	2040	ND	ND	ND
Stott *et al*., 2013 [189]	Human	Oromucosal spray	5	1	0.39	5.28	1.5	3252	22,169	ND	ND
			10	1.39	1.15	6.39	5.0	2546	18,800	ND	ND
			20	1	2.17	9.36	10.4	3783	30,595	ND	ND
Hosseini *et al*., 2021 [12]	Human	Sublingual wafer	25	4.5	9.1	ND	31.1	ND	ND	ND	ND
			50	4.1	15	ND	71.0	ND	ND	ND	ND
		Oral	50	5.2	14	ND	73.8	ND	ND	ND	ND
		Oromucosal spray	25	4.5	4.6	ND	29.3	ND	ND	ND	ND
Meyer *et al*., 2018 [141]	Human	Inhalation	1.6	0.10	7	0.18	2.1	ND	ND	ND	59
		IV	1.6	0.12	22	0.40	13.7	ND	ND	ND	100
Ohlsson *et al*., 1986 [126]	Human	IV	20	0.05	686	24	0.01667 ng·min/L	74.4	2520	ND	ND
		Smoking	19.2	0.05	110	31	0.00485 ng·min/L	ND	ND	ND	31
Devinsky *et al*., 2021 [15]	Human	Oral	50	2.03	6.3	ND	20.05	ND	ND	ND	ND
		Inhalation	2.1	0.06	18.8	ND	7.66	ND	ND	ND	ND
Spindle *et al*., 2020 [135]	Human	Oral (in urine)	100	5.3	776.3	ND	ND	ND	ND	ND	ND
		Vaping (in urine)	100	0.8	261	ND	ND	ND	ND	ND	ND

Abbreviations: AUC, area under the concentration–time curve; CL, clearance; C_max_, peak plasma concentrations; F, bioavailability; MRT, mean retention time; ND, not determined; t_1/2_, elimination half-life; t_max_, time to reach peak plasma concentrations; Vd, volume of distribution

The oral route was used in most clinical studies discussed in this review. However, effective systemic delivery may not be ideal via the oral route due to its low solubility and first pass metabolism. Therefore, understanding the PK profile of different administration routes allows an appropriate choice on CBD delivery to be made that is suitable for specific patients and their medical conditions. Importantly, the different physiology between species can lead to variations in drug responses.

### Oral Administration

Oral delivery is non-invasive, convenient, and the most common administration route for medicines in general [132]. Various oral CBD formulations are available, such as oil drops and oil in capsules [12]. However, oral CBD has demonstrated the lowest bioavailability of 6–19% compared to other delivery routes due to irregular absorption and extensive hepatic metabolism [15, 16, 133]. The high lipophilicity of CBD also contributes to poor and slow absorption as precipitation can occur in the gastrointestinal tract [14]. Studies demonstrated a delayed peak plasma concentration, reached in 2–7 h depending on the dose and subject model, in humans, mice, dogs, horses, and dairy calves [12, 15, 86, 133–136]. The low and delayed absorption into the blood following oral dosing limits CBD delivery to the brain, limiting its activity in acute brain disorders. Despite poor oral absorption, the extent of absorption can be improved by co-administering CBD with a high-fat meal. A 4.85- and 4.2-fold increase in peak plasma concentration (C_max_) and area under the concentration–time curve (AUC), respectively, were observed when compared to the fasting state [137].

The high volume of distribution of CBD indicates its wide distribution throughout body tissues, including the brain, adipose tissue, and other organs. Deiana et al. showed that 120 mg/kg of oral CBD in mice and rats resulted in a C_max_ in the brain of 1.3 µg/mL and 8.6 µg/mL, respectively [138]. CBD was also detected in synovial fluid and joint tissue (articular cartilage and infrapatellar fat pads of stifle joints) in horses and guinea pigs, respectively [139, 140]. These locations of drug distribution support the potential use of CBD in CNS conditions and musculoskeletal pain.

### Intravenous Injection

IV injection is useful in acute indications such as pain, where a rapid, high systemic concentration is required. It bypasses absorption, allowing a 100% drug delivered directly into the systemic circulation as it bypasses the first pass metabolism [86]. Due to the poor solubility of CBD, organic solvents (e.g. ethanol, propylene glycol), surfactants (e.g. Tween 80), polymers (e.g. polyethylene glycol), and oils (e.g. soybean oil, fat emulsion) have been utilized to formulate IV solutions [86, 126, 141, 142]. Xu et al. performed a preclinical study in mice and showed that IV CBD (10 mg/kg) rapidly achieved a high C_max_ of 2343.3 ng/mL (time to reach C_max_ (t_max_) = 0.167 h), which was 18-fold higher than that via the oral route [86]. A lower IV dose was sufficient, thus improving the dosing efficiency. Similarly, Meyer et al. demonstrated that a high drug concentration in humans could be attained within 10 min by IV injection [141]. The fast systemic entry via the IV route allows rapid drug exposure in the brain, and hence may be useful for acute CNS conditions.

Despite the rapid rise in CBD concentration, a significant decline in plasma concentration was observed after IV delivery. Paudel et al. conducted a PK study in rats and showed a 99% reduction in CBD plasma concentration from a C_max_ of 3596 ng/mL to 18.9 ng/mL and 9 ng/mL at 1 and 2 h post injection, respectively. This fast elimination equated to a high clearance of 10.21 L/h/kg and a short elimination half-life of 10 min [142]. Comparable results were shown by Ohlsson et al. in cannabis smokers, in whom a 93% reduction in plasma CBD concentration was observed 1 h after IV administration [126]. More frequent dosing may be necessary to prevent subtherapeutic CBD concentration but the invasiveness of IV route would render administration inconvenient or even impractical for regular treatments.

### Sublingual and Buccal Administration

The sublingual and buccal routes are non-invasive administration methods for avoiding first pass metabolism. Generally, sublingual administration is suitable for rapid systemic drug delivery due to its thin sublingual mucosa (100 – 200 μm) and high vascularization [143]. On the other hand, the moderately vascularized buccal mucosa allows a slower and more predictable drug release so buccal administration is suitable for both local and systemic drug delivery [143]. Various CBD formulations have been developed for these routes of delivery, such as oromucosal spray, sublingual drops, and wafers [12]. CBD can be absorbed through the oral mucous membranes into the systemic circulation and bypass hepatic metabolism [131]. Vitetta et al. found that CBD delivered buccally was quickly absorbed in 1 h but the bioavailability was not reported [144]. The rapid CBD absorption via the sublingual and buccal routes can potentially be effective for emergency use or for fast symptomatic relief, for instance, the use of Sativex oromucosal spray (a 1:1 mixture of CBD and THC) to relieve moderate-to-severe multiple sclerosis spasticity [131]. Absorption via sublingual wafers (t_max_ of 25 vs 50 mg: 4.5 vs 4.1 h) and oromucosal spray (t_max_ = 4.5 h) was faster than that from oral oil administration (t_max_ = 5.2 h) [12].

However, whether sublingual and buccal CBD is absorbed via the oral mucosa or ingested into the gut is still debatable. The washout effect of saliva may contribute to the differences in CBD absorption. A meta-opinion review argued that when the liquid content in Sativex is washed by saliva and swallowed subsequently, a PK profile comparable to that of an oral THC liquid formulation was observed [145]. Therefore, the extent of CBD permeating the sublingual and buccal mucosa should be further examined. The addition of mucoadhesive agents might be useful to prolong drug retention on the mucosa [146].

### Pulmonary Administration

Pulmonary drug delivery is ideal for treating respiratory conditions as it achieves high drug concentration at the target site directly and rapidly [132]. The lungs can also be used for systemic delivery [132]. The respiratory tract epithelium has a total surface area of 100–140 m^2^ and a thickness of 0.2 µm, which provides a large surface area and very thin epithelial layer for rapid and efficient systemic absorption [132]. To deliver drugs efficiently via the lungs, engineering the physicochemical properties of the particles (e.g. aerodynamic particle size, wetting, etc.) is critical. Generally, fine particles with an aerodynamic diameter of 1 to 5 µm are suitable for inhalation into the lungs [147]. Smoked cannabis has a count medium aerodynamic diameter ranging from 0.35 to 0.43 µm [148], which is small enough to be inhaled. However, submicron particles (< 1 µm) tend to be expired rather than depositing in the lungs [147]. On the other hand, a CBD DPI formulation developed by Receptor Life Sciences has a mass median aerodynamic diameter and fine particle fraction of 4.2 µm and 30%, respectively [15], which is suitable for inhalation. Patient factors (e.g. inhalation technique, coughing, etc.) also contribute to the efficacy of inhaled CBD [141, 149]. Spacers are recommended in patients using a pressurized MDIs to improve delivery efficiency [150].

Different forms of pulmonary administration are available such as smoking, vaporization, and inhalation. Smoking is often associated with the recreational use of illicit cannabis because it results in a more rapid onset of action within minutes (t_max_ of smoked CBD = 3 min) with a higher bioavailability than oral (31%) [126]. Although smoking provides almost immediate drug delivery from the lungs to the brain [151], smoking is not preferred clinically due to the generation of toxic by-products from combustion [152]. On the other hand, vaporization demonstrated a similar PK profile as smoked CBD, with a rapid increase in the systemic concentration that peaked much earlier than oral CBD (t_max_ of vaporized vs oral CBD: 0.8 vs 5.3 h) [135]. Vaporization involves heating instead of burning/combusting the herb so it was thought to be relatively safer than smoking [152]. However, as for IV CBD, both smoking and vaping resulted in rapid drug clearance. Ohlsson et al. showed a 90% reduction in the CBD plasma concentration following smoking, from a C_max_ of 110 to 10.2 ng/mL at the first hour [126].

On the other hand, inhalation without heating or combustion is widely used clinically. Common inhalation devices include MDIs, dry powder inhalers (DPIs), and nebulizers [153]. Meyer et al. showed that inhaled CBD via a pressurized MDI and a spacer (8 actuations totalling to 1600 µg CBD) achieved the peak plasma concentration in 6 min, which was comparable to the IV route [141]. The short t_max_ was due to rapid absorption and could potentially be useful for CNS conditions that require rapid symptomatic relief or onset of action. It can also be a good alternative to oral administration to increase CBD bioavailability (59% by inhalation via a MDI) because it bypassed first pass metabolism [141]. Similarly, the increase in CBD bioavailability was demonstrated by Devinsky et al. using a DPI. The dose-adjusted AUC for inhaled CBD was 182.5 ng·h/mL, which was 9.1-fold higher than that from oral administration [15]. The dose-adjusted C_max_ for inhaled CBD was 447 ng/mL, which was 71-fold higher than that obtained with oral CBD. Systemic CBD concentration also peaked earlier (inhaled vs oral t_max_ = 3.8 vs 121.8 min) [15]. In a non-clinical study, CBD was vaporized and detected in the brain of rats (t_max_ = 15 min) but it was mostly eliminated from the brain two hours post-dose [154]. Therefore, rapid lung-to-brain delivery may be possible for treating brain disorders.

### Intranasal Administration

Intranasal administration of CBD has been developed and assessed for its potential treatment in breakthrough pain and nausea [149]. Delivering drugs through the nose is also non-invasive [132]. The nasal cavity has relatively lower enzymatic activity than that in the gastrointestinal tract. In addition, it has a well-vascularized and thin mucosal membrane [155], allowing passive diffusion into the bloodstream and enhancing drug absorption [149, 156]. A recent study in dogs demonstrated a sevenfold faster absorption via the intranasal route compared to the oral route (intranasal vs oral t_max_ = 0.5 vs 3.5 h) [157]. Comparatively, various nasal formulations were developed by Paudel et al. who reported rapid CBD nasal absorption for all the formulations (t_max_ ≤ 10 min) in rats [142]. Intranasal CBD also showed higher bioavailability (34–46%) in an animal study as it avoids drug degradation in the gastrointestinal tract [142]. Previously, all PK studies of CBD via the intranasal route were conducted in animal models, future clinical studies are yet to be conducted to determine the PK profile of intranasal CBD in humans.

The intranasal route allows direct nose-to-brain delivery so it is especially useful for treating neuropsychiatric conditions [158]. Drugs can be absorbed into the brain directly via the olfactory and trigeminal nerve pathways [159]. Although the efficacy of intranasal CBD in CNS conditions has not been confirmed, that of other intranasal drugs has already been demonstrated [160]. For example, intranasal midazolam showed rapid action in reducing pediatric febrile seizures [161]. This supports the future development of intranasal CBD formulations for CNS disorders.

Yet, despite achieving a fast absorption and bypassing hepatic metabolism and blood–brain barrier, drug administered via the nose is subjected to rapid elimination by mucociliary clearance [153]. The disease state of the subjects can also influence the volume of formulation administered. For example, nasal congestion and/or sneezing can lower the dose absorbed [155]. Furthermore, since small volumes of less than 200 µL per dose are typically delivered intranasally, the drug needs to be sufficiently potent [153, 155].

### Transdermal Administration

Transdermal drug delivery plays a significant role in the management of chronic neurological disorders in the geriatric population, such as managing Parkinson's disease, Alzheimer's disease, and neurological pain [162]. Transdermal CBD has successfully achieved sustained pain control in a rat model with chronic inflammatory conditions, such as arthritis [163]. It is non-invasive, as CBD is absorbed from an adhesive patch, cream, or gel through the skin [149]. First pass metabolism is avoided and a sustained steady-state plasma concentration can be achieved over time [156]. This can prevent potential adverse effects associated with the rapid rise in systemic drug levels such as via the IV route. Lodzki et al. reported a significant accumulation of CBD in the skin after the application of 3% w/w CBD patches to the skin of mice [164]. Transdermal application of CBD was also not limited to local distribution as it was also detected in hip skin, abdominal skin, muscles, the liver, and the pancreas in that study [164]. The steady-state plasma concentration of 0.67 µg/mL (about 44% of the initial dose) was attained at 24 h and maintained for at least 72 h [164]. A similar skin reservoir effect was shown in guinea pigs by Paudel et al. [142]. The steady-state CBD level of 6.3 ng/mL was reached at 15.5 h and lasted for 48 h after gel application. It started to decline at about 6 h after gel removal [142]. Achieving sustained therapeutic plasma levels via the transdermal route provides the flexibility of a less frequent dosing regimen in managing chronic brain disorders, subsequently improving patient compliance.

However, for successful transdermal drug delivery into the systemic circulation, and hence through the blood–brain barrier, the drug needs to pass through multiple skin layers, including the lipophilic stratum corneum layer and a deeper hydrophilic skin layer [156]. CBD, being highly lipophilic, can accumulate in the stratum corneum and limit its penetration to deeper skin layers. Bartner et al. carried out a PK study in healthy dogs and showed that the C_max_ of 75 mg and 150 mg CBD-infused transdermal cream were 30.10 and 97.46 ng/mL, respectively, which were 2–8 times lower than the two of their oral formulations [165]. This demonstrated a possible incomplete transdermal absorption due to diffusion barriers.

### Other Routes of Administration

Aside from the routes that were discussed previously in this review, some less common routes may potentially be used for systemic CBD delivery, such as ocular and rectal administration. Although there is so far no study investigating the PK profile of ocular and rectal CBD, these routes for systemic delivery have been shown to be possible for other cannabinoids and other drugs.

There has been increasing interest on ocular CBD for lowering intraocular pressure and its therapeutic potential in treating glaucoma [166–168]. Although the ocular route is mainly for local treatment, systemic absorption from the eyes is possible due to the highly vascularized conjunctiva [132]. Lipophilic drugs such as CBD may be absorbed via the cornea into the aqueous humor, and then into the systemic circulation [169]. Although there is no PK study on ocular CBD yet, the ophthalmic administration of THC in rabbit had been investigated by Chiang et al. [170]. A delayed systemic absorption with a highly variable bioavailability of 6–40% was observed [170]. Due to the chemical similarity of CBD and THC, systemic delivery of CBD via the eyes may be possible but future studies are needed.

Rectal formulations such as suppositories can be used for systemic delivery when oral delivery is not feasible, such as when a patient has dysphagia, nausea, and vomiting [132]. Although first pass metabolism is still possible via the hepatic portal vein into the upper part of the rectum, it can be avoided if the drug is administered to the lower rectum as absorption occurs via the inferior and middle haemorrhoidal veins, which are non-hepatic [132]. An attempt to study the PK profile of 100 mg CBD suppositories in healthy dogs yielded plasma CBD levels that were lower than the limit of quantification, so the data could not be analyzed [157]. Despite that, some studies supported the use of rectal delivery of anticonvulsants, such as diazepam and levetiracetam, for fast and effective seizure treatment [171, 172]. Thus, rectal CBD formulations for epilepsy may be investigated in the future.

## Optimal Routes of Administration

The therapeutic use of CBD is still in its early stages of research for many indications. A wide variety of potential therapeutic effects of CBD has been investigated, particularly on neuropsychiatric conditions. CBD has shown potential benefits in multiple CNS conditions, but more studies are needed to confirm.

Drug administration to the CNS is difficult because it needs to be systemically absorbed first, followed by crossing the blood–brain barrier via passive diffusion. The efficiency of various routes of administration may be evaluated by comparing studies with similar reported nominal doses. An optimal and efficient systemic drug delivery should have a rapid onset of action, high bioavailability, sustained drug effect, flexibility for high dose delivery, and non-invasiveness for self-administration.

Currently, oral and injection routes are the most common formulations for neuropsychiatric drugs, e.g. antiepileptics, analgesics, and antipsychotics. Oral delivery of CBD has been utilized in most preclinical and clinical studies. In fact, the only approved CBD product (Epidiolex) on the market for treatment-resistant epilepsy is delivered orally. Undoubtedly, the oral route is a practical route of drug administration due to its simplicity, convenience, and non-invasiveness. However, the core problems of CBD in its delivery are its low solubility and low oral bioavailability. These limit the drug delivery efficiency and therapeutic potential of CBD, and a higher dose is often required to reach the therapeutic level. Although CBD had been reported to be well-tolerated, with the most prevalent side effects being mild (e.g. tiredness, diarrhea, and changes in appetite) [173], the number and extent of adverse effects may increase with the dose. Various formulations have been investigated to improve the solubility of CBD, such as the use of cyclodextrins [174–176], mesoporous silica [174], polymers [142, 174], self-emulsifying drug delivery systems [177–180], and other nanoformulations [181–183]. By increasing CBD solubility, oral bioavailability can be enhanced. These formulations can also be utilized via other administration routes to increase bioavailability.

Bioavailability can be improved via the injection, sublingual, buccal, pulmonary, intranasal, and transdermal routes because they bypass first pass metabolism. As CNS conditions often require daily dosing to maintain therapeutic drug levels, a non-invasive route that allows easy self-administration is preferred. This can be achieved by intranasal or inhalation delivery as both methods allow non-invasive self-administration. Although transdermal delivery offers sustained steady-state drug concentration, which is ideal for reducing dosing frequency, the absorption is generally slower. The lipophilic property of CBD also restricts it from penetrating into the deeper aqueous skin layer, hence, lowering its bioavailability. On the other hand, despite the rapid drug absorption via the oral mucosa in sublingual and buccal administration, the amount of systemically delivered drug is highly variable. The delivered dose may be affected by saliva wash-out and involuntary swallowing, leading to reduced drug retention time and absorption through the oral mucosa [143]. Unintended ingestion of supposedly sublingual or buccal drugs are subjected to first pass metabolism and food intake [145], thus lowering drug bioavailability.

The nasal mucosa is thin, well vascularized, and has a large surface area for systemic absorption. However, plasma protein binding is a major obstacle against blood-to-brain delivery as only the unbound drug can cross the blood–brain barrier. CBD in plasma, being highly protein bound, can lower its ability to enter and act in the CNS. Yet, the intranasal route bypasses the blood–brain barrier, thus allowing direct nose-to-brain administration via the olfactory and trigeminal nerve pathways. Even though nose-to-brain delivery has gained interest in CNS conditions, an extremely potent drug is necessary as the dose that reaches the brain via the nose is very limited (below 1%) [184]. Considering the CBD dosing in previous evaluated studies was mostly > 5 mg/kg, CBD might not be a potent drug candidate for intranasal delivery. However, novel formulations can be developed to improve CBD solubility, thereby increasing the local concentration of CBD conducive for intranasal administration. Further investigation is required to determine the potency of CBD nasal formulations in brain disorders and the suitability of intranasal delivery in such cases. There is much interest in low dose CBD products and their use is now widespread, although there is very limited evidence for efficacy of CBD at low doses [185].

Inhaled CBD can also achieve faster and higher drug absorption than the transdermal route, owing to the large surface area of the respiratory tract epithelium. This can be useful for treating acute CNS symptoms, such as pain and anxiety attacks. There are currently limited studies on inhaled CBD but interest in this area is increasing. Most of the published studies examined smoking and vaping but these methods are not preferred for clinical use. The safety of the smoked and vaped CBD is questionable as combustion and heating are involved, respectively. Toxic by-products may be produced during these processes. Besides, dose uniformity of these products may not be well controlled.

CBD-only MDI [112, 186] and DPI [15, 187, 188] formulations have been developed and tested in various studies. MDIs can be used with a spacer if needed, while DPIs offer good stability for the drug in solid form. Formulation strategies for CBD dry powders need to overcome the challenges of poor wetting and low solubility, both of which are due to the high lipophilicity of the drug. Aerodynamically small particles should be generated for deep lung deposition to facilitate systemic CBD delivery for neuropsychiatric conditions. Inhaled CBD for treating neuropsychiatric disorders is a growing field and more clinical studies on that are expected to be conducted in the near future.

## Conclusions

There has been increasing interest in, and research on, the potential therapeutic use of CBD due to its non-psychotropic activity. Aside from the approved use of oral CBD (Epidiolex) in treatment-resistant epilepsies, its potential use brain disorders including neuropsychiatric conditions have been explored in preclinical and clinical studies. CNS conditions often require regular dosing, as well as rapid and efficient systemic drug delivery for effective disease management, hence there is a need to find an optimal route of administering CBD. Current CBD treatment via the oral route has delayed absorption and low bioavailability. In contrast, the PK profiles of intranasal and inhalation delivery are superior to those of other routes of administration. They are non-invasive and can elicit fast absorption with increased bioavailability. Although further development and research in this area are required, intranasal and inhalable CBD formulations are potentially of better clinical use for CNS disorders in the future.

## Abbreviations

5-HT: Serotonin
AUC: Area under the concentration–time curve
BCRP: Breast cancer resistance protein
CB: Cannabinoid
CBD: Cannabidiol
CL: Clearance
C_max_: Peak plasma concentration
CNS: Central nervous system
CYP450: Cytochrome P450
DPI: Dry powder inhaler
GPR: G protein-coupled receptor
IV: Intravenous
MDCK: Madin-Darby Canine Kidney II
MDI: Metered dose inhaler
P-gp: P-glycoprotein
PK: Pharmacokinetics
THC: Delta-9-tetrahydrocannabinol
t_max_: Time to reach C_max_
TRP: Transient receptor potential channels
TSC: Tuberous sclerosis complex
Vd: Volume of distribution
